# Hydrogels from a Self-Assembling Tripeptide and Carbon Nanotubes (CNTs): Comparison between Single-Walled and Double-Walled CNTs

**DOI:** 10.3390/nano13050847

**Published:** 2023-02-24

**Authors:** Petr Rozhin, Slavko Kralj, Brigitte Soula, Silvia Marchesan, Emmanuel Flahaut

**Affiliations:** 1 Department of Chemical and Pharmaceutical Sciences, University of Trieste, 34127 Trieste, Italy; 2 Department for Materials Synthesis, Jožef Stefan Institute, Jamova 39, 1000 Ljubljana, Slovenia; 3 Department of Pharmaceutical Technology, Faculty of Pharmacy, University of Ljubljana, Aškerčeva 7, 1000 Ljubljana, Slovenia; 4 Centre Interuniversitaire de Recherche et d’Ingénierie des Matériaux, Université Paul Sabatier, UMR CNRS N°5085, 31062 Toulouse, France

**Keywords:** supramolecular, hydrogels, peptides, self-assembly, carbon nanotubes, composites, nanomaterials, phenylalanine, D-amino acids, nanocarbons

## Abstract

Supramolecular hydrogels obtained from the self-organization of simple peptides, such as tripeptides, are attractive soft materials. Their viscoelastic properties can be enhanced through the inclusion of carbon nanomaterials (CNMs), although their presence can also hinder self-assembly, thus requiring investigation of the compatibility of CNMs with peptide supramolecular organization. In this work, we compared single-walled carbon nanotubes (SWCNTs) and double-walled carbon nanotubes (DWCNTs) as nanostructured additives for a tripeptide hydrogel, revealing superior performance by the latter. Several spectroscopic techniques, as well as thermogravimetric analyses, microscopy, and rheology data, provide details to elucidate the structure and behavior of nanocomposite hydrogels of this kind.

## 1. Introduction

Nanocomposite materials include structures with dimensions in the nanoscale that allow researchers to leverage the exceptional properties of nanomaterials. In recent years, nanocomposites have attracted researchers’ attention in light of their versatile nature, which enables a great variety of applications [1]. The incorporation of anisotropic nanofillers represents a convenient strategy to fine-tune the material properties while attaining nanotechnology-enabled better performance, in comparison with standard alternatives [2]. In this regard, carbon nanomaterials (CNMs) have become popular nanofillers for their exceptional physical and chemical properties [3], which can be modulated ad hoc through chemical derivatization [4,5]. Academic and industrial interest in CNMs has grown thanks to their wide applicability, spanning the diverse areas of catalysis [6,7,8] and energy [9,10,11], pollutant removal [12,13,14,15], aerospace and automotive transport [16,17], targeted drug delivery [18,19], theranostics [20,21], bioimaging [22,23], sensing [24,25,26], regenerative medicine [27,28,29], and flexible electronics [30,31].

In particular, hydrogels are a type of soft matter that benefits from the addition of CNMs to enhance their properties, especially their conductivity, their responsiveness to physicochemical stimuli, and their viscoelastic resistance against applied stresses [32,33,34,35,36]. A plethora of hydrogelators have been studied to develop composites with CNMs; those consisting of minimalistic peptides are widely considered very attractive, thanks to a series of advantageous features. They include their inherent biocompatibility and biodegradability, their benign nature for the environment, their ability to mimic protein functions and display bioactivity [37], and the opportunities that they offer to undergo enzymatic conversion into inactive or active products by design [38]. These building blocks are ideal candidates for the biomimicry of the tissue matrix that is naturally present around cells, and to enable their growth and proliferation for regenerative medicine purposes [39,40,41].

The simpler the peptide sequence, the cheaper will be its production. Therefore, di- and tri-peptides stand out as ideal compounds that can also be produced with cheap liquid-phase synthesis on a large scale [42]. Among them, those featuring the diphenylalanine motif have become very popular for their strong tendency to self-assemble [43,44,45], as well as for their capacity to engage in hydrophobic interactions with CNMs to yield functional composites [46,47,48]. One example is heterochiral D-Leu-L-Phe-L-Phe (or its enantiomer L-Leu-D-Phe-D-Phe), which takes part in Phe zippers thanks to the opposite stereoconfiguration of the N-terminus, relative to the Phe-Phe motif, which effectively positions all the hydrophobic sidechains on the opposite side of the hydrophilic peptide backbone [49]. Such an amphiphilic conformation is a stabilizing feature for the resulting superstructure-based hydrogels [50].

The ability of L-Leu-D-Phe-D-Phe to interact with CNMs to yield hydrogels with enhanced properties was recently investigated. It was found that the addition of oxidized multi-walled CNTs (ox-MWCNTs) yielded hydrogels with the best properties, such as resistance against applied stress relative to graphene oxide (GO) or oxidized carbon nano-horns (ox-CNHs) [51]. Carbon nano-onions [52] and nanodots [53] impede the typical bundling of peptide fibrils into fibers, resulting in more homogeneous hydrogels, but at the expense of a further increase in their viscoelastic moduli. These findings suggest that the anisotropic morphology of CNTs may be an advantageous feature to maximize interactions with peptide fibrils. To confirm this hypothesis and assess the effect of CNT properties on those of the final materials, in this work, we thus sought to investigate the formation of hydrogels with oxidized single-walled CNTs (ox-SWCNTs) or double-walled CNTs (ox-DWCNTs).

## 2. Materials and Methods

### 2.1. Materials and General Methods

Reagents and solvents were purchased from Merck (Milan, Italy) and they were used as supplied, unless indicated differently. All solutions and buffers were prepared using high-purity water, which was obtained from a Milli-Q apparatus (Millipore RiOs/Origin, St. Louis, MS, USA) with a resistivity higher than 18.2 MΩ·cm at 25 °C. The sonicator used to disperse the nanomaterials was the Branson Ultrasonic 3800 (Milan, Italy). SWCNTs were purchased from Nanointegris (super-pure HiPco^®^, lot no. SP2167,1.2–1.7 nm wide, and 0.1–4 µm long).

### 2.2. DWCNT Production

DWCNTs were prepared via catalytic chemical vapor deposition (CCVD) according to a published procedure [54]. They were extracted from the as-produced CCVD material as follows [55]: first, 99.5 mg of DWCNTs was soaked with 40 mL of 4M HCl in a flask with a round bottom. The dispersion was placed in a sonicator for 15 min. Meanwhile, a heating magnetic stirrer was set up at 100 °C and 140 rpm. A round-bottomed flask containing the DWCNTs in HCl (aqua) was set by reflux for 5 h. Filtration of the crude mixture using a Millipore membrane (JHWP, 0.45 μm) was followed by washes with distilled water until neutrality of the filtrate was achieved, then it was washed once with methanol, and once with diethyl ether. The following day, the powder was dried in an oven at 80 °C.

### 2.3. DWCNT Oxidation

DWCNT oxidation was performed in nitric acid [55]. First, 99.5 mg of DWCNTs obtained after the treatment described in Section 2.2 were placed in 100 mL of concentrated HNO_3_ (65% *w*/*w*), sonicated for 15 min at ambient temperature, and then refluxed for 24 h at 130 °C and 300 rpm. Next, the crude mixture was carefully and slowly poured onto ice-cold water (150 mL) for quenching. The mixture was filtered with a Millipore membrane (JHWP, 0.45 μm) and washes were performed with distilled water until neutrality of the filtrate was achieved, then it was washed once with methanol, and once with diethyl ether. The following day the powder was dried in an oven (80 °C).

### 2.4. SWCNT Oxidation

First, 25 mg of super-pure SWCNTs (Sigma-Aldrich, Milan, Italy) was transferred into 50 mL 8M HNO_3_ in a flask with a round bottom, and the reaction was placed in a sonicator at 40 °C for 30 min. The crude mixture was diluted with MilliQ water and filtered with a Millipore membrane (JHWP, 0.45 μm). Washings of the solid material were performed with water until the neutrality of the filtrate was achieved, then it was washed once with methanol, and once with diethyl ether. The following day, the powder was dried in an oven (80 °C).

### 2.5. L-Leu-D-Phe-D-Phe (Lff) Preparation

The tripeptide L-Leu-D-Phe-D-Phe was synthesized using a chlorotrityl chloride resin and Fmoc-protection strategy, isolated by high-performance liquid chromatography (HPLC) in reverse phase, and identified by nuclear magnetic resonance (NMR) and by electrospray ionization mass spectrometry (ESI-MS) coupled to LC, according to previously published procedures [51].

### 2.6. Thermogravimetric Analysis (TGA)

TGA was performed using a TGA5500 instrument (TA Instruments, Milan, Italy) with air or nitrogen gas, as indicated further below in Section 3.1. All samples consisted of 1.0 mg of material. The program used included, first, 20 min of equilibration at 100 °C, followed by a heating ramp at a rate of 10 °C min^−1^ until the temperature reached 800 °C.

### 2.7. Visible-Near Infrared (Vis-NIR) Absorbance Spectroscopy

CNT dispersions were prepared in 1-centimeter quartz cuvettes, using 5 mL *N,N*-dimethylformamide with <0.1 mg of CNTs. Vis-NIR spectroscopy was performed on a Cary 5000 UV-Vis-NIR instrument, using a rate of scanning of 2400 nm min^−1^ and a resolution of 4 nm.

### 2.8. Raman Analysis

Microscopy-grade glass slides were used as the substrate for samples, which were deposited on top of the glass; they were then dried during the night. Raman analysis was performed on ≥5 areas per sample using an inVia 50 instrument from Renishaw (Turin, Italy) and a 785-nm laser (0.25 mW) scanning every cm^−1^.

### 2.9. Transmission Electron Microscopy (TEM)

TEM analyses were performed using a JEM 2100 (Jeol, Tokyo, Japan) at a voltage of 100 kV. Samples were freshly prepared, then they were carefully placed onto TEM grids (carbon-lacey) that had been treated with UV-Ozone Procleaner Plus for 360 s and dried under vacuum. Negative staining was used for contrast (potassium phosphotungstate at 2% with pH 7.2). Micrograph analysis was carried out with ImageJ2 software from FIJI (https://imagej.net).

### 2.10. Fourier-Transformed Infrared (FT-IR) Spectroscopy

FT-IR spectroscopy was performed in attenuated total reflectance (ATR) mode, using a crystal of germanium and applying a resolution of 4 cm^−1^ for 240 scans, on an Affinity-1S instrument (Shimadzu, Milan, Italy).

### 2.11. Self-Assembly into Nanocomposite Hydrogels

First, 2.0 mg of ox-CNTs were poured into a solution (1.0 mL) with 5.0 mg of the tripeptide in sodium phosphate buffer (0.1 M, pH 11.8) and the dispersion was placed in a sonicator for 15 min. Then, another 1.0 mL of slightly acidic sodium-phosphate buffer (0.1 M, pH 5.8) was added to attain a final pH of 7.4, which initiated peptide self-organization into a hydrogel. Samples with 0.1 mg/mL CNTs were prepared accordingly, with a 10-fold reduction in the final amount of CNTs present in the hydrogels.

### 2.12. Oscillatory Rheology

Oscillatory rheology was performed using a Malvern Kinexus Ultra Plus instrument (Alfatest, Milan, Italy) and a substrate consisting of a plate (20 mm) made of stainless steel and featuring parallel geometry. Other parameters included a gap of 1 mm and an ambient temperature (25 °C) that was maintained with a Peltier system (Alfatest, Milan, Italy). Hydrogel samples were freshly prepared on the equipment. Kinetics were monitored at 1 Hz and 1 Pa for 60 min, followed by a sweep of frequency from 10 to 0.1 Hz at a stress of 1 Pa. Finally, stress sweeps were recorded from 1 Pa until 100–200 Pa at 1 Hz.

## 3. Results and Discussion

### 3.1. SWCNT and DWCNT Characterization and Oxidation

**Figure 1:**
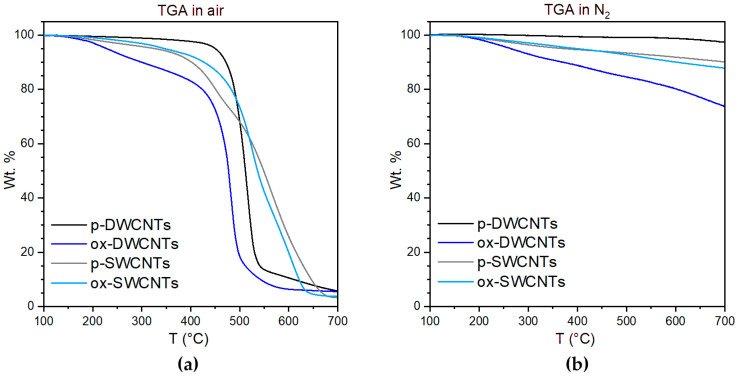
Thermogravimetric analysis (TGA) of pristine (p-) and oxidized (ox-) CNTs (**a**) in air and (**b**) in nitrogen.

SWCNTs and DWCNTs were first oxidized with nitric acid to favor their dispersibility in aqueous buffers compatible with tripeptide self-assembly. Thermogravimetric analysis in air (Figure 1a) of the pristine CNTs revealed a superior quality of the DWCNTs (black) relative to SWCNTs (grey), with DWCNTs displaying a higher graphitic content with <3 wt % amorphous carbon, a sharper transition in air at 485 °C, and a residue of 5 wt % at 700 °C. TGA in nitrogen gas (Figure 1b) was used to monitor the level of CNT functionalization, revealing, for oxidized DWCNTs, a 24 wt % loss at 700 °C relative to the pristine material, and corresponding to 5.6 mmol COOH/g considering carboxylic acids as the main type of functional group being introduced via the process. In contrast, the level of oxidation for SWCNTs was significantly lower (i.e., 2.7 wt % loss at 700 °C, relative to pristine SWCNTs, corresponding to 0.63 mmol COOH/g). We inferred that even in mild conditions, the oxidized CNTs were severely damaged; those remaining after the washings displayed a minimal content of oxygen-containing functional groups.

**Figure 2:**
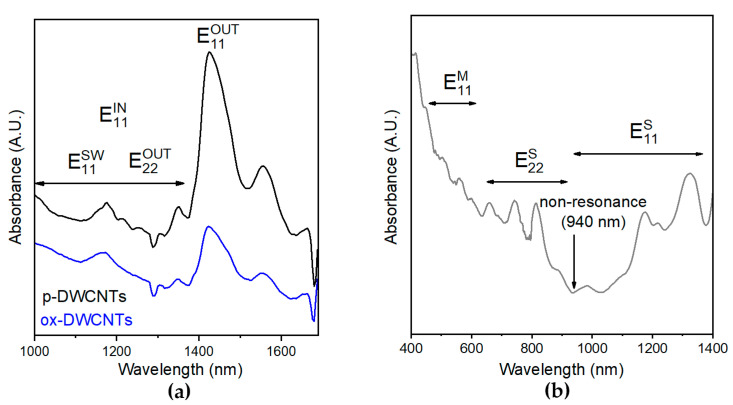
Absorbance spectra of CNTs. (**a**) NIR spectra of pristine (p-) and oxidized (ox-) DWCNTs. (**b**) Vis-NIR spectrum of untreated SWCNTs.

Visible near-infrared (Vis-NIR) absorbance was also assessed to characterize the CNTs. In particular, the NIR spectra of DWCNTs (Figure 2a) displayed a wide signal at 1425 nm that was ascribed to the outer walls’ first optical transition of semiconducting CNTs (E_11_^OUT^). The signals that were present between 1000 and 1370 nm were mainly associated with the corresponding transition of the inner walls of semiconducting DWCNTs (E_11_^IN^), as well as to the analogous transition of SWCNTs (E_11_^SW^), and to the DWCNT outer walls’ second optical transition (E_22_^OUT^). The latter two signals have been reported to be weaker than the first one, and this observation can also be ascribed to lower amounts of SWCNTs. After oxidation, the E_11_^OUT^ peak was significantly reduced in intensity, while the rest of the spectrum did not change significantly. Considering that the loss of optical response can be ascribed to the grafting of chemical functionalities, we inferred that the oxidation occurred only on DWCNTs’ outer walls while maintaining the integrity of the interior walls. The modest reduction in magnitude noted for the remaining signals was rationalized as being due to the minor contributions of E_11_^SW^ and E_22_^OUT^, which we envisaged would be impacted by oxidation [56].

The Vis-NIR spectrum of untreated SWCNTs is displayed in Figure 2b; it confirmed their semiconducting nature, with the absence of a signal due to E_11_ transitions for the metallic SWCNTs in the range of 440–635 nm. Conversely, the E_11_ and E_22_ transitions for the semiconducting SWCNTs were visible in the ranges of 940–1400 nm and 630–940 nm, respectively [57]. The spectrum of the oxidized SWCNTs was not significantly different (see the Supplementary Materials, Figure S1), confirming a minimal extent of functionalization.

**Figure 3:**
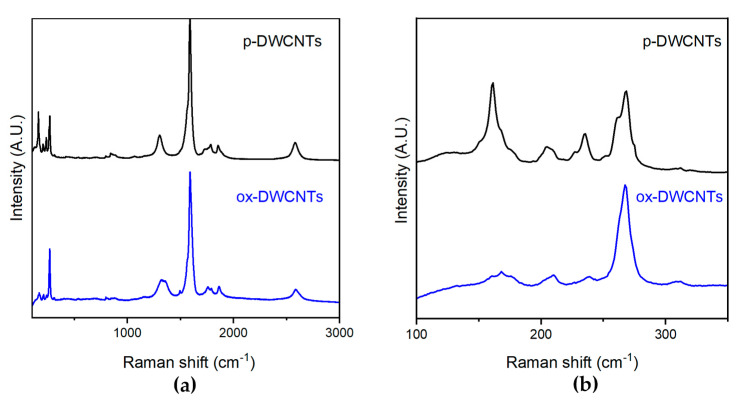
Raman spectra of pristine (p-) and oxidized (ox-) CNTs: (**a**) full-range spectra; (**b**) RBM region to calculate DWCNT diameters (see).

Raman analyses were then carried out to assess the level of defects of different CNTs [58]. In the case of SWCNTs, a minor extent of oxidation was confirmed with an increase in the intensity ratio between the so-called D band and the G band (I_D_/I_G_) from 0.16 ± 0.05 to 0.24 ± 0.04 (see Supplementary Materials Figures S2 and S3). In the case of DWCNTs (Figure 3a), I_D_/I_G_ increased from 0.19 ± 0.06 to 0.27 ± 0.05, indicating successful covalent functionalization, resulting in an increase in defects on the graphitic surface.

The radial breathing mode (RBM) region (Figure 3b) of the DWCNTs’ spectra displayed several peaks that arose from all the radial vibrations of carbon atoms of the DWCNTs. Their corresponding frequencies ω (cm^−1^) were related to the CNTs’ diameters, *d* (nm), in an inverse proportion, established by the following equation:

ω = α/*d* (1)

with α = 238 cm^−1^ as an average value that considered the van der Waals interactions between the DWCNTs’ interior and exterior walls, which was similar in nature to those occurring between tubes within SWCNT bundles. Furthermore, RBM frequencies, ω, above 200 cm^−1^ were associated with the interior walls, in agreement with the observation of DWCNTs’ minimal interior diameter being 0.40 nm, with a distance between carbonaceous walls corresponding to 0.34 nm [59]. The calculated DWCNTs diameters are shown in Table 1. In the case of SWCNTs, negligible differences were found in the intensities, rather than the frequency, of the RBM signals (see the Supplementary Materials, Figure S3, and Table S1), from which an average diameter *d* = 1.0 ± 0.1 nm was calculated [60].

**Figure 4:**
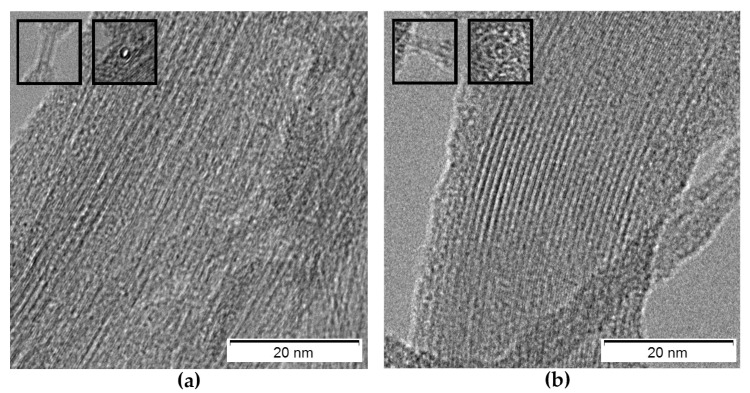
TEM images of (**a**) pristine (p-) and (**b**) oxidized (ox-) DWCNTs. Insets show details of individual DWCNTs crossing the focal plane, where *d* ~ 1.6 nm.

TEM micrographs revealed the occurrence of bundles of DWCNTs before and after the oxidation process (Figure 4), with diameters in agreement with those calculated by Raman spectroscopy. For comparison, SWCNTs displayed larger amounts of amorphous carbon (see Supplementary Materials, Figure S4).

**Figure 5:**
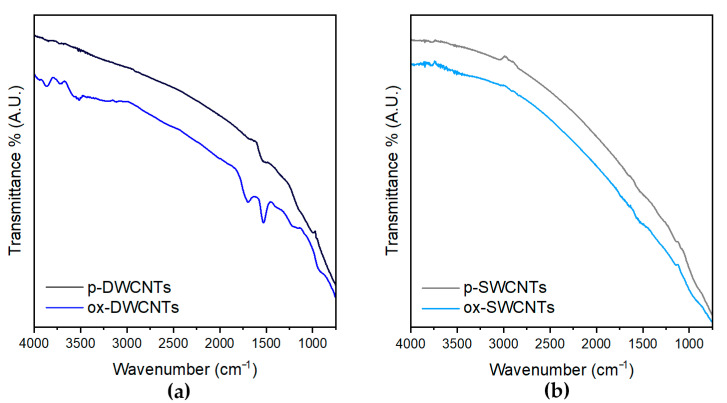
FT-IR spectra of pristine (p-) and oxidized (ox-) CNTs: (**a**) DWCNTs; (**b**) SWCNTs.

Fourier-transformed infrared (FT-IR) spectroscopy was used to assess the oxidation of CNTs (Figure 5) [61]. This technique further confirmed the introduction of COOH functionalities in DWCNTs (Figure 5a), with the rising of the typical carbonyl stretching signal at 1702 cm^−1^, and of the weak C-O signals at 1220 cm^−1^, and several signals in the region of 3000–3800 cm^−1^, where OH stretchings of COOH and OH groups, along with water, are located. Finally, the C=C signal at 1535 cm^−1^ that was also present in the pristine DWCNTs increased in intensity after oxidation [62]. In agreement with the limited oxidation of SWCNTs observed with the other techniques, the FT-IR analysis revealed negligible differences between pristine and oxidized SWCNTs (Figure 5b).

### 3.2. Hydrogels with Self-Assembling Tripeptide and CNTs

**Figure 6:**
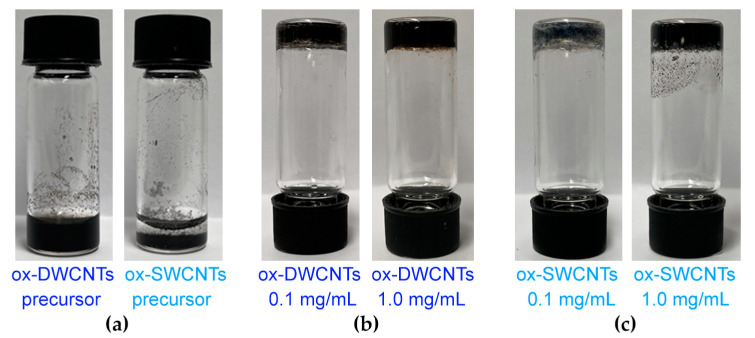
Photographs of peptide and CNTs in sodium phosphate solutions. (**a**) Gel-precursor alkaline solutions with peptide and CNTs. (**b**,**c**) Self-supporting hydrogels were obtained with peptide and (**b**) ox-DWCNTs or (**c**) ox-SWCNTs at a neutral pH.

The tripeptide L-Leu-D-Phe-D-Phe was produced using a chlorotrityl chloride resin and Fmoc-protection strategy, purified by HPLC in reverse phase, and characterized spectroscopically, as previously described [51]. In the typical self-assembly protocol, a solution of the hydrophobic tripeptide is obtained at a basic pH so that it is present as an anion, which does not self-assemble due to the repulsion between the negative charges. Subsequent pH lowering to neutral values yields the zwitterions that establish ionic interactions between the charged termini, to enable peptide stacking and self-organization into gelling superstructures [49]. When oxidized CNTs were added to the gel precursor solution of the peptide at alkaline pH, remarkable differences were noted between the DWCNTs and SWCNTs (Figure 6a). In particular, a homogenous dispersion of ox-DWCNTs was obtained even at concentrations as high as 1 mg/mL, while ox-SWCNTs precipitated even when diluted ten times more (i.e., 0.1 mg/mL, Figure 6a). However, the subsequent lowering of pH to neutrality triggered self-assembly in all cases, yielding self-supporting hydrogels (Figure 6b,c). Nevertheless, those obtained with ox-SWCNTs were not homogenous, with evident segregation between the CNTs and peptide (Figure 6c). We inferred that although CNTs did not impede peptide self-organization, even at high loadings of 40% *w*/*w* relative to the L-Leu-D-Phe-D-Phe, the ox-SWCNTs’ tendency to aggregate was remarkably higher than that of ox-DWCNTs. We attributed the difference to the extent of grafting of the polar functional groups on their surface since the length and diameter between the tubes were comparable.

**Figure 7:**
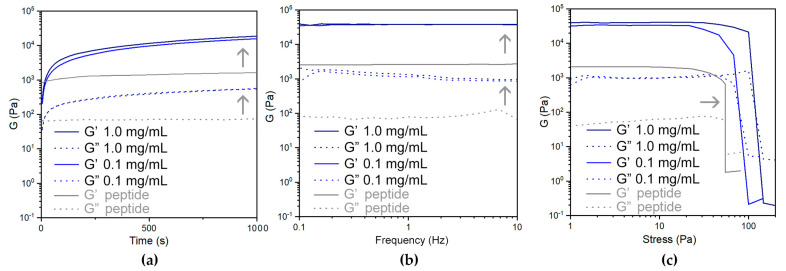
Oscillation-based rheometry of peptide hydrogels without (grey) or with (blue) ox-DWCNTs: (**a**) kinetics; (**b**) frequency ramps; (**c**) stress ramps. Grey arrows indicate ox-DWCNTs’ effects on the viscoelastic properties of the peptide gels.

**Figure 8:**
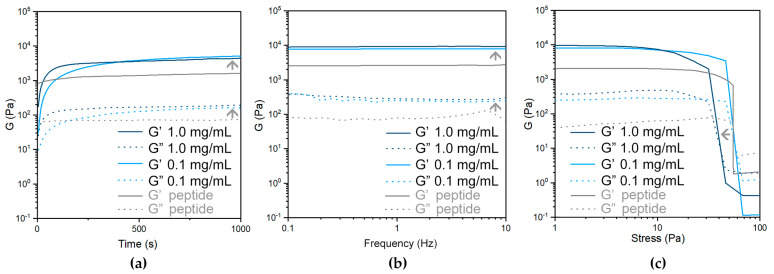
Oscillation-based rheometry of peptide hydrogels without (grey) or with (blue) ox-SWCNTs: (**a**) kinetics; (**b**) frequency ramps; (**c**) stress ramps. Grey arrows indicate ox-SWCNTs’ effects on the viscoelastic properties of the peptide gels.

The viscoelastic character of the materials was investigated by rheometry, using oscillation analyses (Table 2 and Figure 7 and Figure 8). Time sweeps revealed fast gelation kinetics within seconds (Figure 7a and Figure 8a), confirming the visual observations. In the case of ox-DWCNTs (Figure 7a), hydrogels reached an elastic modulus (G’) of 38 kPa within an hour, regardless of the CNT loading (i.e, 0.1 or 1.0 mg/mL), with nearly a 20-fold increase relative to the peptide alone [51]. Frequency sweeps confirmed the hydrogel’s nature, with the elastic (G’) and the viscous (G”) moduli both being not dependent on the oscillation frequency (Figure 7b). Then, stress ramps revealed a significant increase in the resistance of the gels’ integrity [51], with a gel rupture occurring at 110 Pa when the ox-DWCNTs were loaded at 1.0 mg/mL.

Within an hour, the hydrogels with ox-SWCNTs (Figure 8a) reached a 4-fold higher elastic modulus G’ relative to the peptide alone. Such change in G’ was comparable to that resulting from the inclusion of ox-MWCNTs [51], and significantly lower than that obtained with ox-DWCNTs (Figure 7). Frequency sweeps confirmed the hydrogel character, with neither the G’ nor G” moduli being dependent on the oscillation frequency (Figure 8b). Surprisingly, stress ramps (Figure 8c) did not reveal an increase in the stress required to rupture the gels; rather, the opposite was found for higher loadings of ox-SWCNTs (i.e., 1.0 mg/mL).

**Figure 9:**
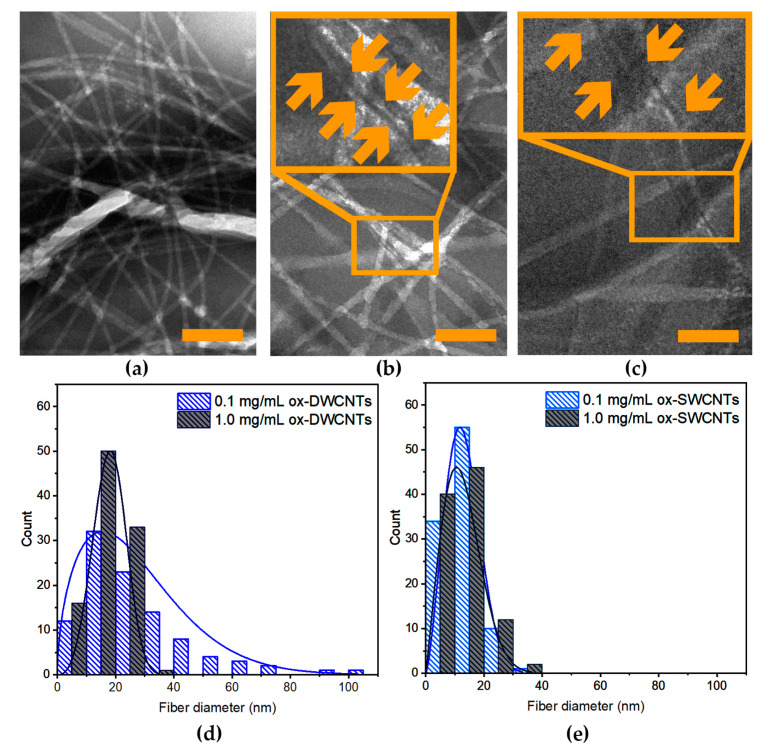
TEM analysis of composite hydrogels. (**a**–**c**) TEM micrographs of gels (**a**) without CNTs, and with 0.1 mg/mL (**b**) ox-DWCNTs or (**c**) ox-SWCNTs. Scale bars = 100 nm. (**d**,**e**) Peptide fibers’ diameter distributions for samples with (**d**) ox-DWCNTs and (**e**) ox-SWCNTs.

TEM analyses were performed to shed further light on the composite hydrogels (Figure 9 and Supplementary Materials, Figures S5–S8). All samples displayed a network of peptide fibrils and fibers (shown in white in Figure 9a–c), with instances of CNTs connecting them (black in Figure 9b,c and indicated by orange arrows in the insets). Statistical analyses of the peptide fibers’ diameter distribution (Figure 9d,e) revealed significantly wider fibers in the case of samples with ox-DWCNTs, relative to those with ox-SWCNTs, in agreement with higher elastic moduli for the former, registered with rheometry. Specifically, the median values corresponded to 22.2 ± 19.1 nm and 19.2 ± 5.6 nm for samples with 0.1 and 1.0 mg/mL ox-DWCNTs, respectively, and to 12.9 ± 5.7 nm and 11.4 ± 6.4 nm for samples with 0.1 and 1.0 mg/mL ox-SWCNTs, respectively. Clearly, those samples with higher loadings of ox-DWCNTs displayed a significantly narrower distribution of the peptide fibers’ diameters, relative to those with lower loadings of ox-DWCNTs (Figure 9d), effectively resulting in less bundling and more connecting points in the fibrillar network. This could explain the higher resistance against applied stress that was noted with rheology for the former sample. In contrast, no significant difference was found in fibers’ diameters for samples with lower or higher loadings of ox-SWCNTs (Figure 9e), which was in agreement with their analogous viscoelastic behavior in the rheological time and frequency sweeps (Figure 8a,b). However, instances of significantly shorter fibrils were found for the samples with 1.0 mg/mL ox-SWCNTs (see Supplementary Materials, Figure S8), which could explain the lower resistance against applied stress noted for this sample (Figure 8c).

## 4. Conclusions

The design of composite hydrogels based on the supramolecular organization of short peptides and CNMs is not trivial. The effects of changing properties from one CNM to the other are still being elucidated. Specifically, an analogous elongated morphology of CNTs and L-Leu-D-Phe-D-Phe assemblies is useful to improve the viscoelastic properties of these hydrogels. CNT oxidation is key to mitigating their strong tendency toward aggregation. However, striking the right balance is challenging for SWCNTs, since chemical oxidation comes at the expense of their structural integrity, leading to significant sample loss. Conversely, DWCNTs are an attractive alternative type of nanomaterial that allows to introduce polar groups on their outer walls, while preserving the inner walls [63]. Furthermore, they enhanced the viscoelastic properties of the peptide gels, performing better than ox-MWCNTs or ox-SWCNTs in terms of the extent of the increase in the elastic modulus. In the future, it will be interesting to explore the electronic properties of these materials [64] and their potential biological applications, for instance, as scaffolds for the regeneration of conductive tissues [65].

## Figures and Tables

**Table 1:** DWCNTs diameters (*d*) derived from the RBM frequencies (ω) [59].

p-DWCNTs		ox-DWCNTs	
ω (cm^−1^)	*d* (nm)	ω (cm^−1^)	*d* (nm)
150	1.6	-	-
161	1.5	161	1.5
168, 175	1.4	168, 175	1.4
204	1.2	203	1.2
209	1.1	211	1.1
227, 235	1.0	238	1.0
251, 261, 268, 275	0.9	267	0.9
310	0.7	310	0.7

**Table 2:** Viscoelastic properties of the peptide hydrogels without and with ox-CNTs.

Hydrogel Material	CNT Loading (mg/mL)	G’ (kPa)	G” (kPa)	Gel Rupture (Pa)
Peptide hydrogel [51]	0	2.0 ± 0.2	0.1 ± 0.0	70 ± 14
+ ox-DWCNTs	0.1	38 ± 5.5	1.0 ± 0.1	72 ± 11
+ ox-DWCNTs	1.0	38 ± 2.8	1.0 ± 0.1	110 ± 19
+ ox-SWCNTs	0.1	8.2 ± 2.4	0.3 ± 0.1	63 ± 6.7
+ ox-SWCNTs	1.0	7.6 ± 2.8	0.4 ± 0.1	45 ± 23
+ ox-MWCNTs [51]	0.1	3.0 ± 1.0	0.1 ± 0.0	110
+ ox-MWCNTs [51]	1.0	6.1 ± 2.0	0.2 ± 0.1	250

## Data Availability

Data are available in the Supplementary Materials and from the authors upon reasonable request.

## Supplementary Materials

The following supporting information can be downloaded at: https://www.mdpi.com/article/10.3390/nano13050847/s1, Figure S1: Vis-NIR absorbance spectra of SWCNTs; Figure S2: Raman spectra of SWCNTs; Figure S3: RBM range of Raman spectra of SWCNTs; Figure S4: TEM micrographs of SWCNTs; Figures S5–S8: TEM micrographs of composite gels; Table S1: SWCNT diameters calculated form RBM frequencies.
