# Supplementary material for: Hydrogels from a Self-Assembling Tripeptide and Carbon Nanotubes (CNTs): Comparison between Single-Walled and Double-Walled CNTs
Source: Nanomaterials (Basel). 2023 Feb 24;13(5):847. doi: 10.3390/nano13050847 (PMC10005271; doi:10.3390/nano13050847)
Supplement: Supplementary file 1 [file nanomaterials-13-00847-s001.zip › nanomaterials-2236983-supplementary.pdf]

**Supplementary Materials**

# Hydrogels from a Self-Assembling Tripeptide and Carbon Nanotubes (CNTs): Comparison between Single-Walled and Double-Walled CNTs

**Petr Rozhin [1], Slavko Kralj [2,3], Brigitte Soula [4], Silvia Marchesan [1,*] and Emmanuel Flahaut [4,*]**

[1] Department of Chemical and Pharmaceutical Sciences, University of Trieste, 34127 Trieste, Italy
[2] Department for Materials Synthesis, Jožef Stefan Institute, Jamova 39, 1000 Ljubljana, Slovenia
[3] Department of Pharmaceutical Technology, Faculty of Pharmacy, University of Ljubljana, Aškerčeva 7, 1000 Ljubljana, Slovenia
[4] Centre Interuniversitaire de Recherche et d'Ingénierie des Matériaux, Université Paul Sabatier, UMR CNRS N°5085, 31062 Toulouse, France
* Correspondence: smarchesan@units.it (S.M.); emmanuel.flahaut@univ-tlse3.fr (E.F.)

## Table of Contents

1. Vis-NIR spectra of SWCNTs…………………………………………………………….. 2
2. Raman spectra of SWCNTs and diameter calculation………………………………… 2
3. TEM micrographs of SWCNTs………...……………………………………………….. 4
4. TEM micrographs of composite gels………………………………………………….. 4

## 1. Vis-NIR spectra of SWCNTs

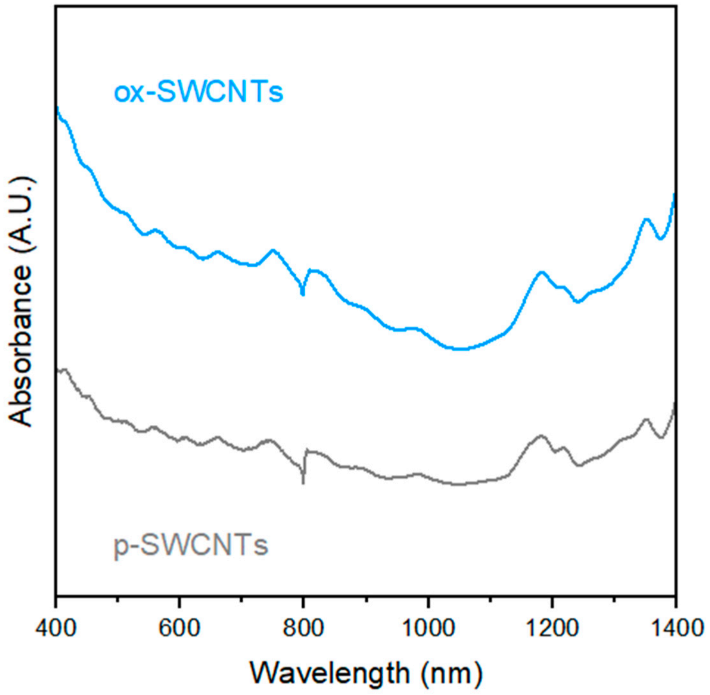


**Figure S1.** Vis-NIR absorbance spectra of pristine SWCNTs (p-SWCNTs, grey) and oxidized SWCNTs (ox-SWCNTs, light blue). No significant difference was found between the two simples.

## 2. Raman spectra of SWCNTs

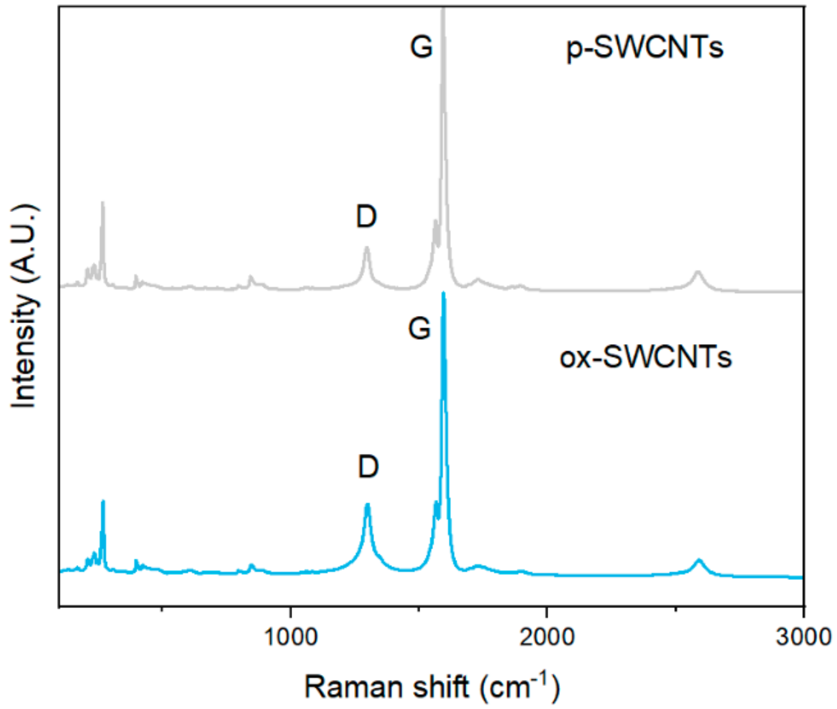


**Figure S2.** Raman spectra of pristine (p-) SWCNTs (grey) and oxidized (ox-) SWCNTs (light blue).

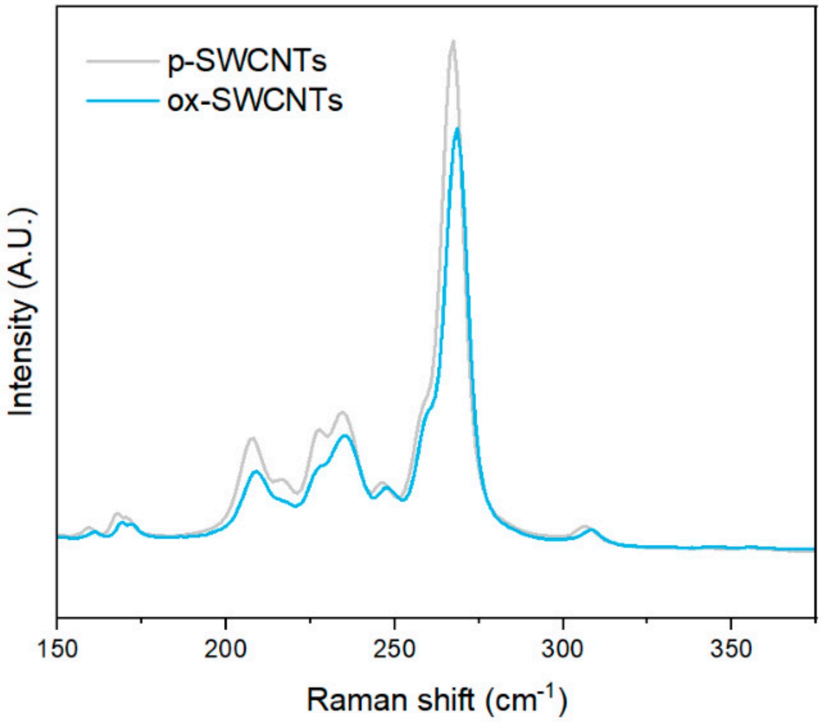


**Figure S3.** RBM region of Raman spectra of pristine (p-) SWCNTs (grey) and oxidized (ox-) SWCNTs (light blue).

**Table S1.** SWCNT diameters (*d*) calculated from the RBM frequencies (ω), using the equation $\omega_{RBM} = A/d_t + B$, where A is associated with the vibrational force constant of the $sp^2$ C–C bond, and B is related to environmental effects. For typical SWCNT bundles, A = 234 $cm^{-1}$·nm and B = 10 $cm^{-1}$ (see S. Costa et al., Materials Science-Poland 2008, 26, 433). The average calculated SWCNT diameter was 1.0 ± 0.1 for both pristine and oxidized SWCNTs.

| **p-SWCNTs** | | **ox-SWCNTs** | |
|---|---|---|---|
| **ω ($cm^{-1}$)** | ***d* (nm)** | **ω ($cm^{-1}$)** | ***d* (nm)** |
| 208 | 1.2 | 208 | 1.2 |
| 217, 228 | 1.1 | 226 | 1.1 |
| 235, 247 | 1.0 | 235, 248 | 1.0 |
| 267 | 0.9 | 269 | 0.9 |

## 3. TEM micrographs of SWCNTs

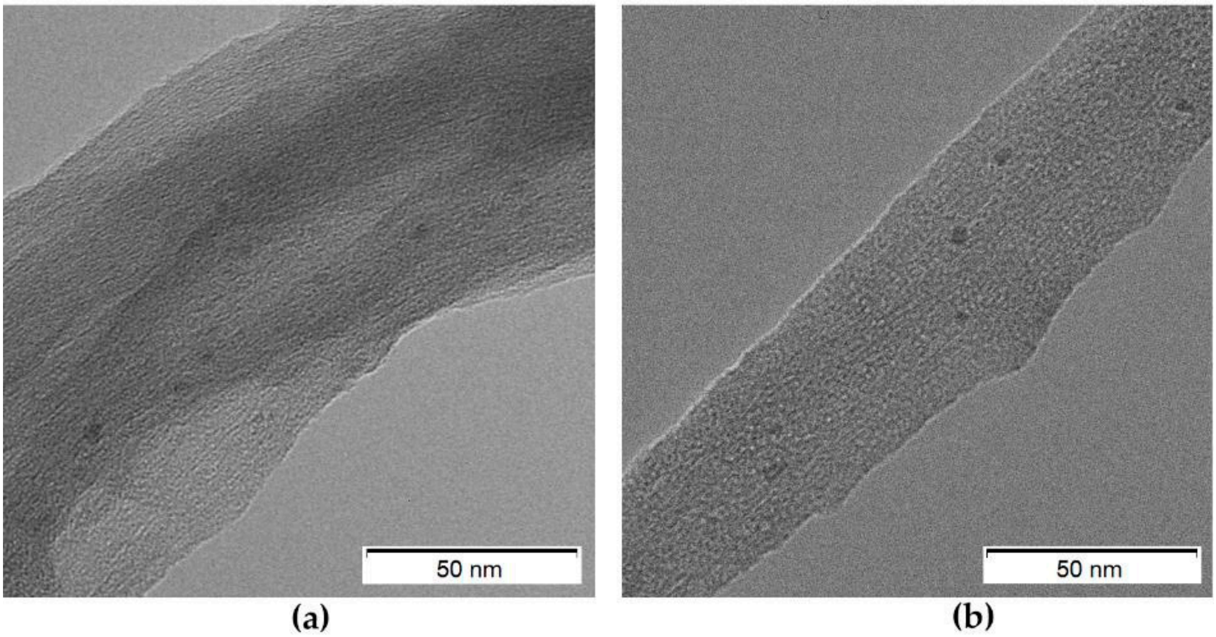


**Figure S4.** TEM micrographs of **(a)** pristine SWCNTs and **(b)** oxidized SWCNTs.

## 4. TEM micrographs of composite gels

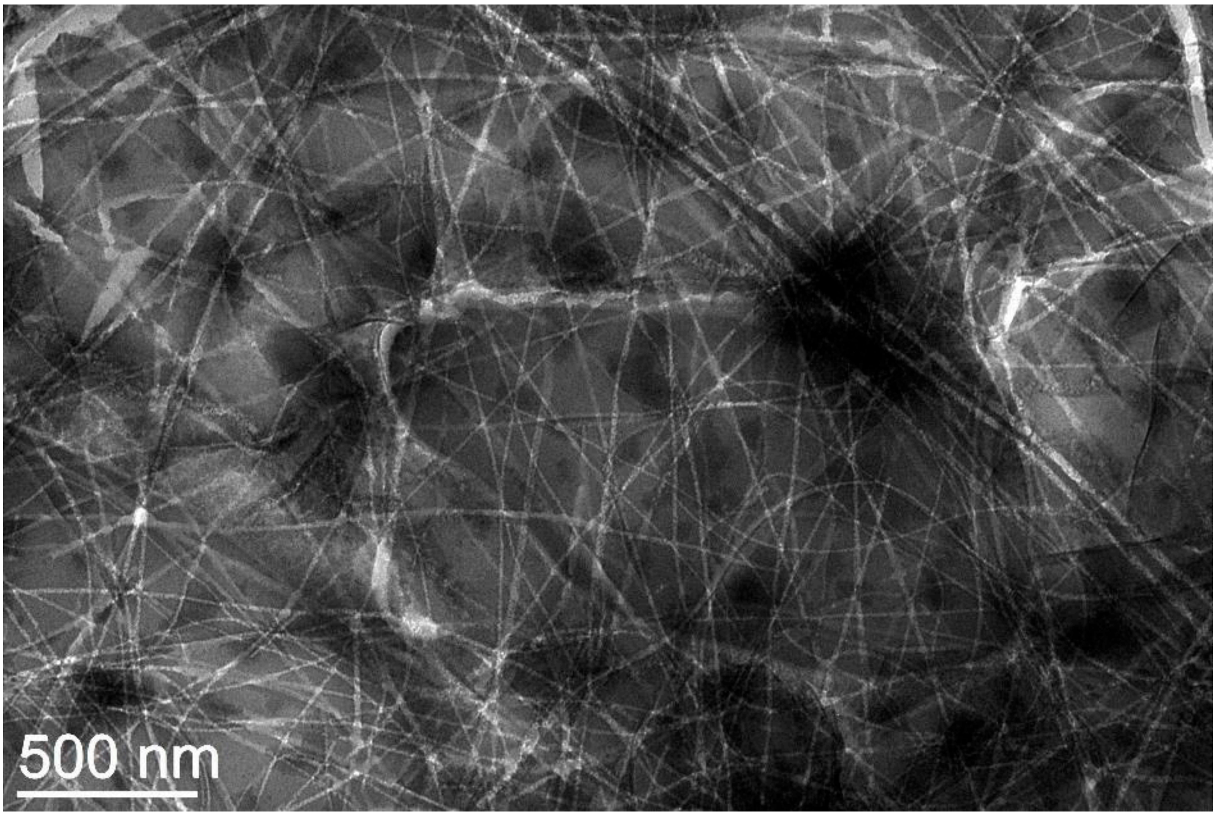


**Figure S5.** TEM micrograph of composite gel with 0.1 mg/mL ox-DWCNTs.

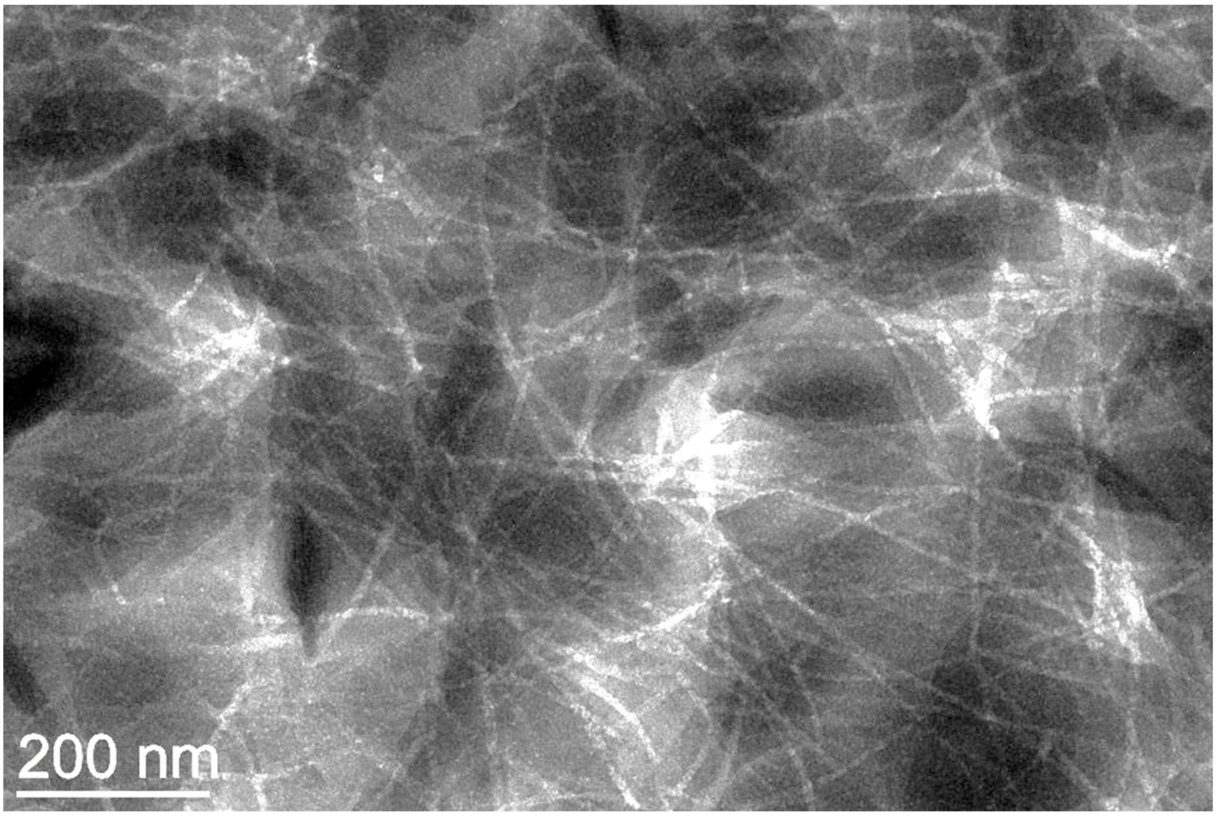


**Figure S6.** TEM micrograph of composite gel with 1.0 mg/mL ox-DWCNTs.

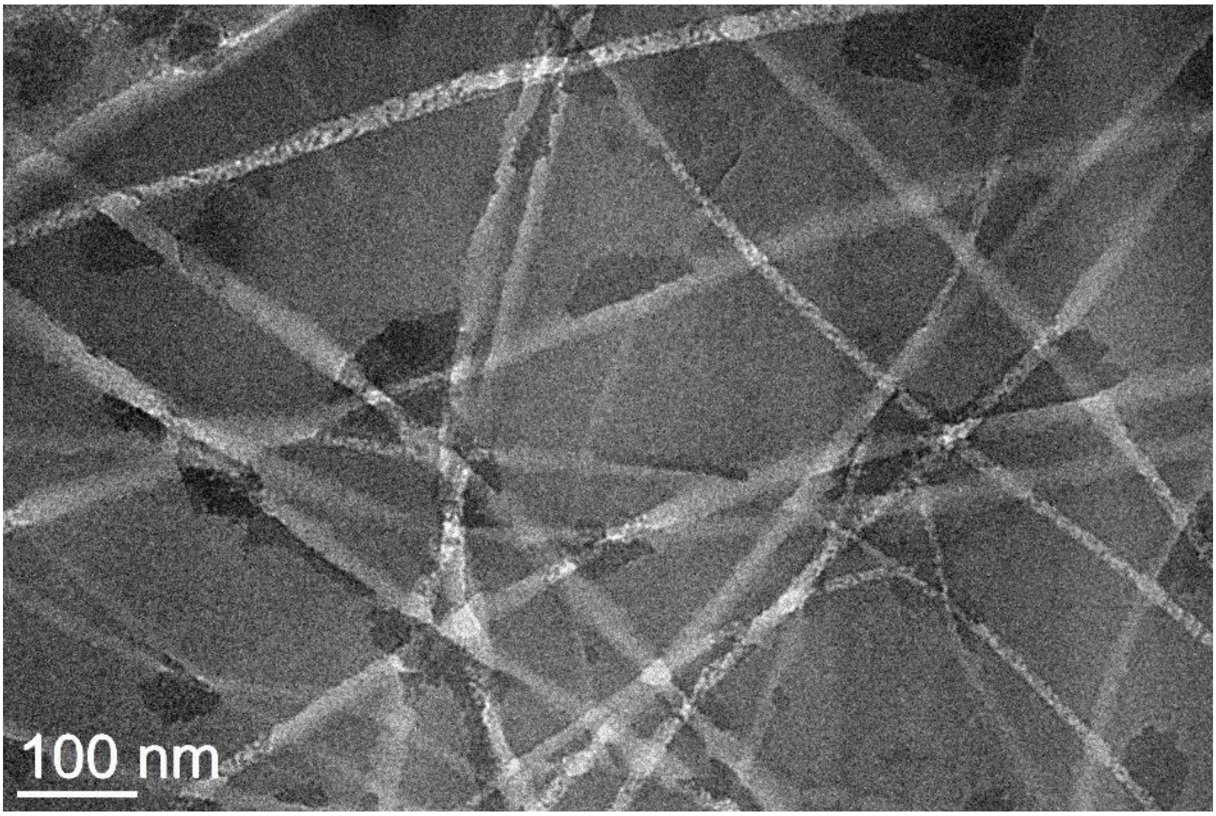


**Figure S7.** TEM micrograph of composite gel with 0.1 mg/mL ox-SWCNTs.

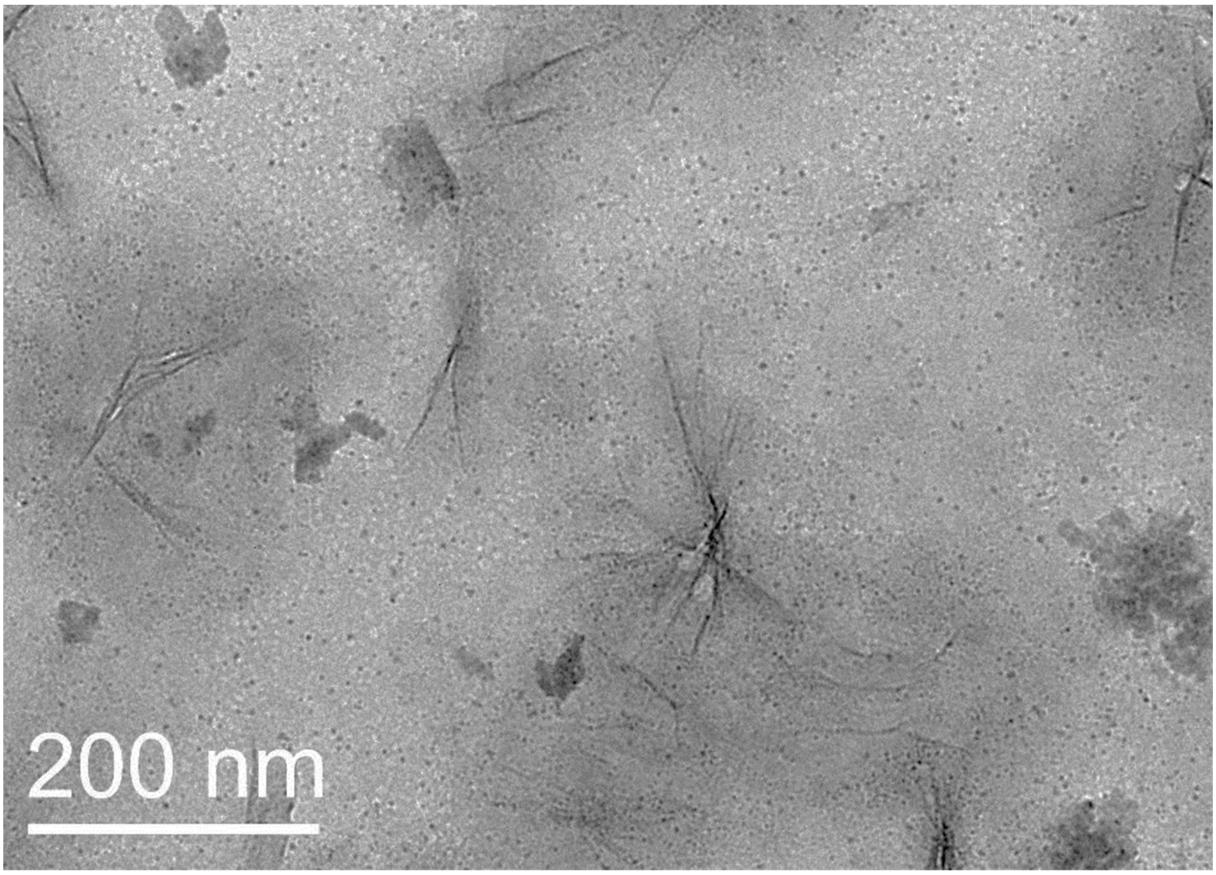


**Figure S8.** TEM micrograph of composite gel with 1.0 mg/mL ox-SWCNTs.
